# Open wedge high tibial osteotomy: cause of patellar descent

**DOI:** 10.1186/1749-799X-7-3

**Published:** 2012-01-12

**Authors:** Jason CH Fan

**Affiliations:** 1 Department of Orthopaedics & Traumatology, Alice Ho Miu Ling Nethersole Hospital, Hong Kong SAR, People's Republic of China

## Abstract

This was a retrospective review of the nine open wedge high tibial osteotomy (HTO) done in a regional hospital in Hong Kong from 2006 to 2008. The mechanical hip-knee-ankle angle improved from average 169.5°(164°-175°) to average 183.9° (179°-187°). Patellar descent was noted in all patients postoperatively, with Blackburne-Peel (BP) index significantly changing from 0.78 (0.64-0.93) to 0.59 (0.38-0.78) (p < 0.05). This change was strongly correlated with the size of anterior bone graft (r = -0.766; p = 0.016). The patellar tendon length as measured by Insall-Salvati index was not changed (pre-operative: 1.02 (0.88-1.25), final: 1.09 (0.8-1.22) (p = 0.683)), inferring that scarring contracture of patellar tendon was not related to patellar descent.

## Introduction

High tibial osteotomy (HTO) has been well described as an effective procedure for treatment of medial compartmental osteoarthritis of knee, especially in young and active individuals. It could be done with barrel-vault osteotomy as decribed by Maquet [1], or with closing-wedge osteotomy as suggested by Coventry [2], or with open-wedge osteotomy as described by Hernigou [3]. The last one can be performed on the medial side without any muscle detachment from the anterior tibial compartment. The fibula and proximal tibiofibular joint are left intact, and therefore the risk of peroneal nerve injury is minimal. The correction in both frontal and sagittal planes can be fine-tuned intraoperatively [4]. The resultant tibio-condylar offset is the least [5].

Patella baja is a well known complication after HTO. Kaper et al reported that 54% of 46 knees that had undergone lateral closing-wedge HTO had lowering of patellar height of greater than 10% [6]. It also occurred in open-wedge HTO. Actutally, Brouwer et al showed that more patella baja was created after an open-wedge HTO than after a closing-wedge HTO [7].

The aim of this study was to discuss the cause of patellar descent after open-wedge HTO. The clinical outcome of the patients and the technical tips to achieve satisfactory result would also be explained.

## Materials and methods

Between July 2006 to August 2008, nine open-wedge HTO in six patients were done. They included four female and two male patients. The mean age was 49 (37-53). Five right knees and four left knees were operated. The inclusion criteria were medial joint line pain and tenderness and minimal lateral compartment and patellofemoral joint symptom. The knee flexion contracture was less than 10° and flexion range was greater than 90°. Radiograph of knee showed varus deformity and predominant narrowing of medial joint space while the lateral and patellofemoral joint spaces were preserved. Exclusion criteria included tricompartmental osteoarthritis, inflammatory arthritis, complete collateral deficiency, poor knee range outside that stated above in the inclusion criteria and age older than 65 year-old. Moreover, patients with significant tenderness of patellofemoral joint (PFJ) associated with greater than 50% of PFJ narrowing shown in skyline radiograph of knee were also excluded. Marginal osteophyte and cruciate ligament deficiency were not regarded as exclusion factors.

**Figure 1:**
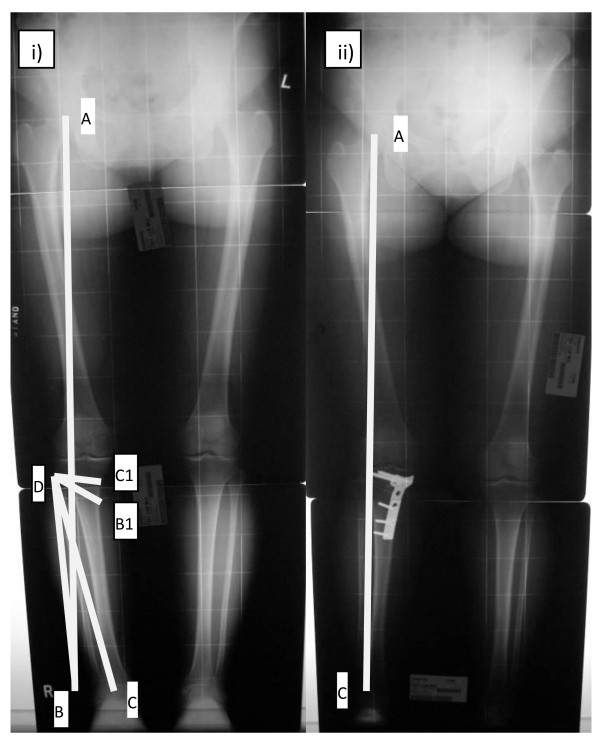
**i) Preoperative planning**. A: femoral head center; AB: Fujisawa line; C: ankle center; D: Pivot point. Tibial shaft tracing distal to DC1 rotated laterally until point C lying on line AB, DC1 intersected medial tibial cortex to form point B1. C1B1 was the size of posteromedial bone graft. ii) Postoperative radiograph. Line AC intersected the knee joint at 30% lateral to knee center.

Preoperative planning was done in a standing scanogram (Figure 1). The planned osteotomy cut in the frontal plane was determined by connecting a point 3 cm below the medial joint line and another point just above the fibular head. Fujisawa line was drawn by connecting femoral head center and another point at 30% lateral to center of tibial plateau surface [8]. Next, a tracing of the portion of tibia distal to the osteotomy was made. It was then superimposed on the radiograph and rotated around the lateral pivot until the ankle center lied on the Fujisawa line. The amount of medial opening was marked and measured. The new mechanical hip-knee-ankle angle was then measured to make sure that it was about valgus three to six degrees as suggested by Hernigou et al [3].

Operation was done using longitudinal incision about 1 cm medial to the patella tendon. The pes anserinus was cut and part of the distal attachment of medial collateral ligament was stripped. The space superior to the tibial tuberosity (TT) and deep to the patellar tendon was developed. One Kirschner wire was inserted from medial side along the line connecting TT upper border and the upper end of proximal tibiofibular joint under x-ray control. Lateral x-ray was taken to locate the osteotomy plane parallel to the joint surface. Another Kirschner wire was inserted at that plane parallel to the first wire under x-ray control. Osteotomy was performed distal to the wires initially by an oscillating saw, and then with osteotome, which stopped at about 5 mm from lateral cortex, under x-ray control. Another osteotome was stacked onto the first one to gradually spread the space open. Bone spreader was inserted to open it further. Diathermy cable was placed along the line connecting hip and ankle centers under x-ray control. X-ray of knee confirmed the cable passing through the Fujisawa point and there was no lateral translation of distal segment. The open space at the most posteromedial aspect was measured, which was the size of the corner tricortical bone graft. While ensuring full knee extension, the sizes of anterior and middle bone spaces were measured. Three pieces of tricortical bone graft were harvested from the ipsilateral iliac crest, and then impacted into corresponding places. 4.5 mm T-plate was used to fix the osteotomy. Knee range of motion was tested before wound closure. The knee was mobilized one day after operation. Six weeks of non-weight bearing walking and another six weeks of partial weight bearing walking were advised for healing of bone graft.

Postoperative assessment was undertaken at about three months and latest follow-up. Mechanical hip-knee-ankle (HKA) angle and knee joint obliquity (Ob) were measured in standing scanogram. Insall-Salvati (IS) index [9], Blackburne-Peel (BP) index [10] and posterior tibial slope as measured by Moore and Harvey (MH) method [11] were obtained in lateral knee radiograph in at least 30° flexion.

From the medical records, the preoperative and the latest clinical data were obtained, including the bone graft size, follow-up period, knee range, clinical symptom and Knee Society knee score [12]. Hernigou et al reported that the best result of HTO was to achieve mechanical HKA between 183° and 186° [3]. Knee society knee score was modified in the alignment part. If the HKA was between 183° and 186°, zero point was deducted. From 174° to 182° and from 187° to 195°, two points was deducted for every degree beyond the ideal range. For the other alignment, 20 points was deducted. Student t-test was used to compare preoperative, 3-month and latest value of HKA, Ob, IS index, BP index and MH slope. The relationship between different variables was calculated by linear regression method, and reported in Pearson's correlation (r) and significance (p). The Statistical power analysis for the correlation between anterior bone graft (ABG) size, posterior bone graft (PBG) size, BP change and IS change was done by computer software GPOWER.

## Results

The mean follow-up period was 19.6 months (11-36). The sizes of anterior and posterior bone grafts were 5.4 mm (4-7) and 16.3 mm (11-20) respectively. Table 1 showed the preoperative, 3-month and latest data of IS index, BP index, mechanical hip-knee-ankle angle, joint obliquity and MH slope. Besides IS index and MH slope, when comparing the 3-month data and the latest data with the preoperative data, the p-values were all < 0.05, indicating statistically significance. Preoperative IS index was similar to those at 3-month and in the latest follow-up (p = 0.463 and p = 0.3). MH slopes before operation were similar to those at 3-month and in the latest follow-up (p = 0.872 and 0.536). For the comparison of all variables between 3-month data with the latest data, all differences were statistically insignificant.

**Table 1:** Data indicating the patella tendon length (IS index), patella position related to joint surface (BP index), Coronal knee alignment (HKA and Joint Ob) and sagittal alignment (MH slope).

Mean(range)	Preoperation	3-month	Latest Fu
**IS index**	1.02 (0.88-1.25)	1.06 (0.9-1.28)	1.09 (0.8-1.22)

**BP index**	0.78 (0.64-0.93)	0.55 (0.42-0.67)*	0.59 (0.38-0.78)*

**HKA (°)**	169.5 (164-175)	184.4 (179-188)*	183.9 (179-187)*

**Joint Ob (°)**	Var 5.5 (2-10)@	Val 7.3 (2-13)*	Val 7.7 (4-11)*

**MH slope (°)**	10.9 (5-15)	10.6 (4-18)	11.8 (8-16)

Var: varus; Val: valgus. Positive value of MH slope indicated posterior slope.

* p < 0.05 when compared with preoperative data. @ p = 0.023 when compared with normal var 3°.

In case of intact patellar tendon, IS index actually measured the ratio of patellar tendon length to the diagonal length of patella, which thus cancelled the effect of radiographic magnification. While BP index measured the ratio of perpendicular distance of lower edge of patellar articulation from the tibial plateau to length of patella articular surface, it could better reflect the patellar height. The effect of sizes of anterior and posterior bone graft, and MH Slope on the patellar tendon length and patellar height was separately correlated. Only the size of anterior bone graft (ABG) was significantly but inversely correlated with change of patellar height (r = -0.766; p = 0.016). The posterior bone graft (PBG) size and change of MH slope were not correlated with change of BP index. For the change of patellar tendon length, the correlations of ABG, PBG and change of MH slope were not statistically significant (Table 2).

**Table 2:** Linear regression reported the Pearson's correlation (r) and significance (p value) of the relationship between anterior bone graft (ABG) size, posterior bone graft (PBG) size, Moore-Harvey (MH) slope change and Blackburne-Peel (BP) index change and Insall-Salvati (IS) index change.

	ABG size	PBG size	MH slope change
**BP change**	r = -0.766	r = -0.122	r = 0.204

	p = 0.016*; P = 1	p = 0.755; P = 1	p = 0.599

**IS change**	r = -0.474	r = -0.111	r = -0.261

	p = 0.198; P = 1	p = 0.775; P = 1	p = 0.498

MH Change, BP change and IS change were calculated by preoperative data subtracted from latest data. Statistical power (P) was calculated by GPOWER.

* Statistically significant

There was no flexion contracture before and after operation. The preoperative flexion was 123.3°(110°-125°) and the postoperative flexion was 118.3°(100°-125°)(p = 0.22). The function score improved from 57.8(50-90) to 80(45-100), and the knee score increased from 45.4(35-52) to 87.1(70-100). They were statistically significant (p < 0.05). The final function and knee scores were not correlated with the latest BP index (r = -0.214, p = 0.58; r = 0.036, p = 0.928 respectively).

One patient had displacement of posterior bone graft and loss of corrected alignment at one week after operation. Reoperation with bone graft reposition and internal fixation was necessary. There was no complication of wound infection, hematoma, compartment syndrome, intraoperative fracture of tibial plateau fragment or neurovascular injury.

## Discussion

The average longevity of a HTO has been estimated to be about six to seven years, with conversion to total knee arthroplasty (TKA) occurring in about 20% of patients after 5 years, 40% after 10 years, and 60% after 15 years [6]. Patella baja, change of tibial slope and valgus alignment after HTO can all cause technical difficulties during TKA, particularly in relation to eversion of patella, exposure of the lateral compartment, and placing the tibial component in the correct position both for rotation and slope [7].

In the present study, patella descent occurred in all knees early after HTO, with the BP index decrease from 0.78 to 0.55. The patellar height was maintained until the latest follow-up, with BP index of 0.59. The BP indexes of three knees (0.38, 0.44 & 0.49) were outside normal range (0.54-1.06) and were defined as patella baja. The patella tendon length was insignificantly increased early after operation. It was maintained until the latest follow-up. This pointed to a structural cause of patellar descent, rather than a biological cause due to scarring. Moreover, the size of ABG was found to be significantly but inversely related to the BP index change. The larger the ABG, the larger was the patella descent, the smaller was the latest BP index, and the more negative would be the BP change. Rigid fixation and early mobilization in the present cases could minimize the patellar tendon scarring. This echoed the idea of Brouwer et al that elevation of the tibial plateau after open-wedge osteotomy was one of the cause of patella baja [7]. Alteration of tibial slope was reported to affect the patellar height [6,7]. It could not be concluded in the present study (r = 0.204, p = 0.599). Moreover, the patellar height was not found to be correlated to the final functional and knee score.

Tibial slope in the current study was reported by the method of Moore and Harvey [11] because lateral radiograph of the whole tibia was not available to draw the mechanical tibial axis. Moreover, it was reproducible in the lateral knee radiograph by using the anterior tibial cortex and tibal plateau surface as landmarks. The posterior tibial slope was not significantly altered after HTO. It was maintained after complete union of bone graft in the latest follow-up. A few factors contributed to the success. Osteotomy cut was confirmed by x-ray to be parallel to the tibial plateau surface. The knee was kept in full extension when measurement of bone graft sizes was done, and also during the fixation of osteotomy by plate. Lateral x-ray of the contralateral knee was obtained to compare the tibial slope. If there was pre-existing knee flexion contracture and the osteotomy was used to correct that deformity, the posterior tibial slope would be diminished.

Over-correction of coronal valgus alignment was important to obtain satisfactory result after HTO [3,13]. Hernigou et al reported that the best results were obtained in 20 cases of open-wedge HTO that had mechanical hip-knee-ankle angle of 183° to 186° [3]. There was no pain and no progression of the arthrosis in either the medial or the lateral tibiofemoral compartment. Aglietti et al reported the results of 91 closing-wedge HTO [13]. An anatomical valgus alignment at consolidation between 8° and 15° was significantly correlated with the best result. In the current study, the former recommendation was followed because it was more stringent and open-wedge HTO was performed. In this way, the Knee Society knee score was also modified because the target range in HTO was different from that in TKA. The mechanical coronal alignment was significantly corrected from varus 10.5° to valgus 4.4°. At consolidation of bone graft, it was maintained at valgus 3.9°. The varus recurrence was minimal and not significant. Six out of nine knees could achieve the target alignment. One knee had varus 1° and the patient had minimal knee pain and his knee score was 86 at 24 month follow-up. Two knees had valgus 7° and the patients had no knee pain. One of their knee scores was 98 at 36 month follow-up and the other was 87 at 27 month follow-up. In order to achieve the target alignment, a few points were important, including accurate preoperative planning, careful prevention of lateral tibial translation on opening up the osteotomy and measurement of bone graft size, harvesting graft larger than measured size to allow down-trimming and rigid internal fixation.

The normal knee joint has varus 3° obliquity. The preoperative value of the present cases was varus 5.5° (2°-10°) (p = 0.023). It indicated significant medial compartment bone loss. It was changed to valgus 7.7°(4°-11°) at bone graft consolidation. One knee had valgus 11°. Hernigou et al found that their maximum postoperative obliquity was 10° [3]. After follow-up of eleven years, no medial subluxation of tibia was noted.

The knee range of motion was not altered significantly after HTO, while the function and knee scores were significantly improved. Only one patient had deterioration of function score after HTO. His function score was 60 before his left HTO, which ameliorated his left knee pain. He had exacerbation of right knee pain and the function score was 55 before his right HTO at about eight months later. In the most recent follow-up at 22 months after first HTO, the function score decreased to 45. The latest HKAs were 183° and 185° and the knee scores changed from 47 and 45 to 75 and 70 for right and left sides respectively. He had exacerbation of bilateral patellofemoral joint pain related to increasing osteoarthritic change, which affected the knee score. In addition, he had bilateral calves pain related to spinal problem, which affected his functional status. This last case exemplified the importance of patient selection in achieving good postoperative result. Even with ideal alignment, postoperative knee pain exacerbation and functional deterioration could happen.

## Conclusion

Patellar descent was common after open-wedge HTO. It was found to be correlated with the size of anterior bone graft. In order to achieve satisfactory coronal and sagittal alignment, it was important to avoid the pitfalls and adhere to some of the technical details. Meticulous patient selection was also important in order to ensure that the good postoperative result could be maintained.

## Competing interests

The author declares that they have no competing interests.

## Authors' contributions

JCHF carried out the operation, did the data collection and analysis and wrote the manuscript.
